# FBXW7 suppresses epithelial-mesenchymal transition, stemness and metastatic potential of cholangiocarcinoma cells

**DOI:** 10.18632/oncotarget.3355

**Published:** 2015-01-31

**Authors:** Hui Yang, Xiaofei Lu, Ziming Liu, Lili Chen, Yunfei Xu, Yuli Wang, Guangwei Wei, Yuxin Chen

**Affiliations:** ^1^ Department of Hepatobiliary Surgery, Qilu Hospital of Shandong University, Jinan, China; ^2^ Department of General Surgery, Jinan Central Hospital of Shandong University, Jinan, China; ^3^ Department of Emergency Medicine, Jinan Fifth People's Hospital, Jinan, China; ^4^ Department of Pathology, Jinan Fourth People's Hospital, Jinan, China; ^5^ Department of Anatomy and Key Laboratory of Experimental Teratology, Ministry of Education, Shandong University School of Medicine, Jinan, China

**Keywords:** ubiquitin ligase, tumor suppressor, mTOR, ZEB1, metastasis

## Abstract

Epithelial-mesenchymal transition (EMT) plays a fundamental role in cancer metastasis. The ubiquitin ligase FBXW7, a general tumor suppressor in human cancer, has been implicated in diverse cellular processes, however, its role in cholangiocarcinoma (CCA) metastasis has not been identified. Here, we report a crucial role of FBXW7 in CCA metastasis by regulating EMT. Loss of FBXW7 expression was detected in CCA cells and clinical specimens. Clinicopathological analysis revealed a close correlation between FBXW7 deficiency and metastasis, TNM stage and differentiation in intrahepatic CCA and perihilar CCA. Moreover, FBXW7 silencing in CCA cells dramatically promoted EMT, stem-like capacity and metastasis both *in vitro* and *in vivo*. Conversely, FBXW7 overexpression attenuated these processes. Mechanistically, treatment with rapamycin, a mTOR inhibitor, inhibited EMT, stem-like capacity and metastasis induced by FBXW7 silencing both *in vitro* and *in vivo*. Furthermore, the expression of EMT regulating transcription factors, snail, slug and ZEB1, were also decreased markedly with rapamycin treatment. In addition, silencing ZEB1 inhibited EMT and metastasis of both CCA cells and FBXW7 deficient CCA cells, which implicated the potential role of ZEB1 in FBXW7/mTOR signaling pathway related CCA metastasis. In conclusion, our findings defined a pivotal function of FBXW7 in CCA metastasis by regulating EMT.

## INTRODUCTION

Cholangiocarcinoma (CCA), the second most common primary hepatobiliary malignancy, is an epithelial cell malignancy originating from the bile ducts. The most contemporary classification based on anatomical location includes intrahepatic (IHCC), perihilar (PHCC), and distal (DCC) CCA [1]. The incidence and mortality rate of CCA are increasing worldwide [2]. However, 5-year survival for CCA patients is disappointing, the median survival is only 15 months [3] and more than two thirds of CCA patients are diagnosed with advanced stage. Therefore, dissecting the molecular mechanisms of CCAs is urgent for identifying early diagnosis and effective chemotherapy markers for CCA patients.

FBXW7 (or hCdc4), a member of the F-box family of proteins, is a substrate recognition component of the Skp1-Cul1-F box protein (SCF) ubiquitin ligase complex [4, 5]. It has been shown to mediate the ubiquitin-dependent proteolysis of several well-known oncoproteins, including Notch, cyclin E1, mammalian target of rapamycin (mTOR), c-Myc, and c-Jun [6]. FBXW7 governs diverse cellular processes, including cell-cycle progression, cell proliferation, differentiation, DNA damage response, maintenance of genomic stability, and neural cell stemness [6]. Generally, FBXW7 is regarded as a tumor suppressor in human cancers. However, the function of FBXW7 in tumor metastasis is rarely reported. Akhoondi et al. [7] have reported the mutant frequency of FBXW7 is 35% in twenty CCA patients, but whether it plays a role in CCA metastasis remains unknown.

Metastasis is an essential factor correlated with poor prognosis of CCA patients [3, 8]. Increasing evidence revealed tumor metastasis was correlated with epithelial to mesenchymal transition (EMT) [9]. The process of EMT involves profound phenotypic changes that include the loss of cell-cell adhesion, cell polarity and the acquisition of migratory and invasive properties and the mesenchymal state is believed to associate with the capacity of cells to migrate to distant organs and maintain stemness, allowing their subsequent differentiation into multiple cell types during development and the initiation of metastasis [9]. However, few studies have investigated EMT mechanisms in CCA [10-12]. Thus, uncovering the regulatory mechanisms of EMT should provide greater insight into the signaling programs that govern metastasis in CCA.

With respect to these notions, the present study first investigated the clinical significance of FBXW7 in CCA. The role and underlying mechanisms of FBXW7 in regulating EMT and metastasis of CCA cells were also explored. Our results not only further elucidated the metastasis mechanism of CCA but also identified FBXW7 may serve as a potential molecular marker for advanced CCA treatment.

## RESULTS

### FBXW7 expression deficiency correlates with IHCC and PHCC metastasis

**Figure 1:**
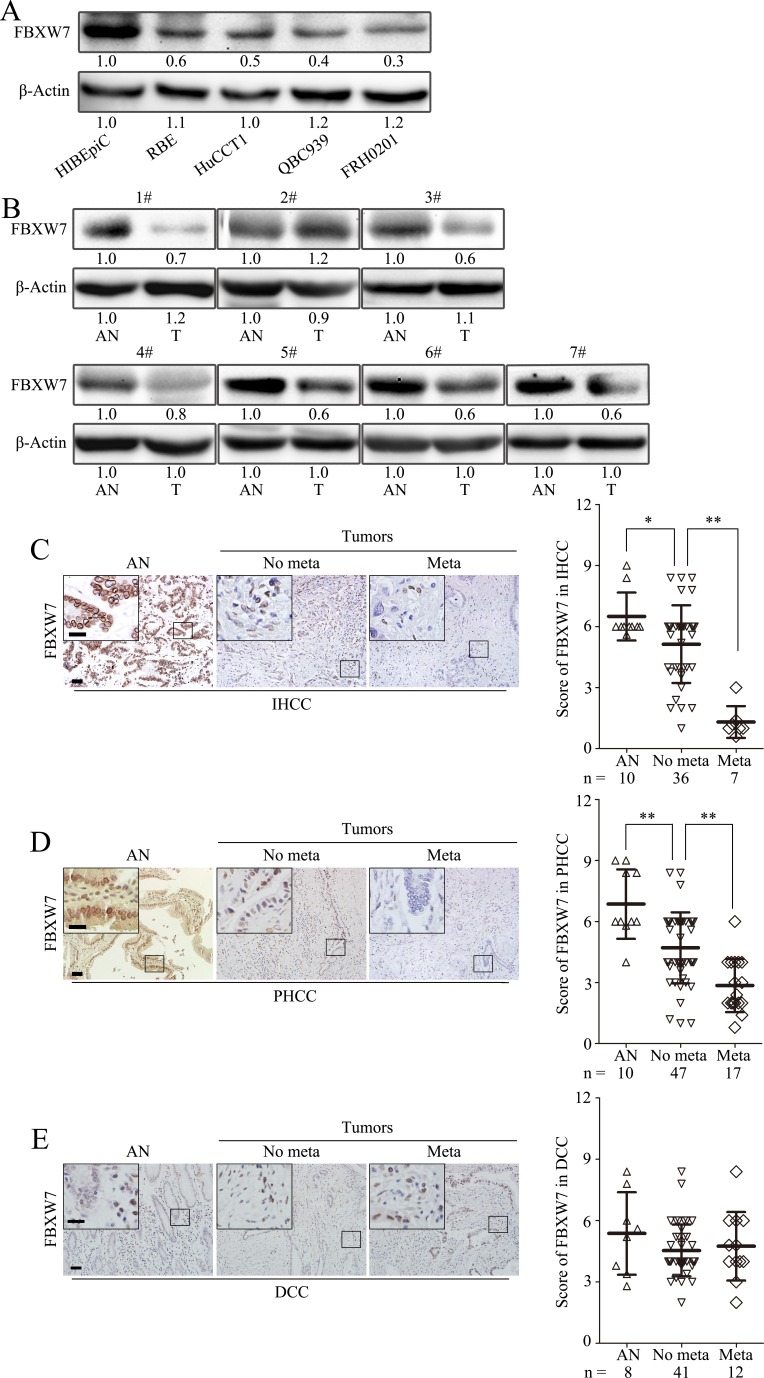
FBXW7 expression deficiency correlates with CCA metastasis A. FBXW7 expression was analyzed by Western blotting in HIBEpiCs and four human CCA cell lines. B. The expression of FBXW7 in 7 paired samples of CCA tissues versus adjacent normal bile duct tissues was measured by Western blotting. AN, Tumor adjacent nontumorous tissue; T: Tumor. C, D and E. Representative images of FBXW7 IHC staining in IHCC (C), PHCC (D) and DCC (E) primary cancer tissues with or without metastasis and corresponding tumor adjacent nontumorous tissues. Corresponding semiquantification of FBXW7 expression was shown in right panels. AN, Tumor adjacent nontumorous tissue; No meta, primary cancers without metastasis (in situ); Meta, primary cancers with metastasis. Scale bars: 50μm (C, D and E) and 20μm (insets in C, D and E). Numbers in (A and B) indicate the fold changes of band densities based on at least three independent experiments. * *P* < 0.05 and ** *P* < 0.01 based on the Student *t* test. Data are represented as mean ± SD.

To investigate the clinical significance of FBXW7 in CCA development, the expression of FBXW7 in normal human intrahepatic biliary epithelial cells (HIBEpiC) and four human CCA cell lines was firstly analyzed. Western blotting and qRT-PCR analyses showed that FBXW7 level was lower in CCA cells than in HIBEpiCs (Fig. 1A; Supplementary Fig. S1A). Reduced FBXW7 protein level was also found in six of seven primary CCA tumors relative to adjacent nontumorous bile duct (Fig. 1B; Supplementary Fig. S1B), implicating FBXW7 may also be a tumor suppressor in CCA as reported in other types of tumors [7]. To verify our observations and define the clinical significance of FBXW7 in CCA, immunohistochemical (IHC) staining was performed in a cohort of 160 CCA specimens (43 IHCC, 64 PHCC and 53 DCC), 28 of them with paired tumor adjacent nontumorous tissues (10 intrahepatic bile duct tissues, 10 perihilar bile duct tissues and 8 distal bile duct tissues). IHC staining confirmed the downregulation of FBXW7 in cancer tissues compared with tumor adjacent nontumorous tissues in IHCC and PHCC, but not in DCC (Fig. 1C-E). Most importantly, correlation analysis of FBXW7 protein levels with clinicopathologic features revealed significant association between deficiency of FBXW7 expression and metastasis, TNM stage and differentiation in IHCC and PHCC (Fig. 1C-E; Table 1). These data suggest that FBXW7 expression is downregulated in CCA and negatively correlates with metastasis, TNM stage and histological grade of human IHCC and PHCC, indicating the involvement of FBXW7 deficiency in IHCC and PHCC progression.

**Table 1:** Correlation Between FBXW7 Expression and Clinicopathological Characteristics of CCA Tumors

Clinicopathological parameters	IHCC(n=43)			PHCC(n=64)			DCC(n=53)		
	n	IHC score	*p*	n	IHC score	*p*	n	IHC score	*p*
Primary tumor			*P*<0.01^a^			*p*>0.05^a^			p>0.05^a^
	<5CM 17	5.71±1.80		<3CM 39	4.16±1.93		<3CM 45	4.64±1.29	
	≥5CM 26	3.85±2.28		≥3CM 25	4.31±1.69		≥3CM 8	4.27±1.73	
Tumor with Metastasis			*P*<0.01^a^			*P*<0.01^a^			p>0.05^a^
No	36	5.14±1.91		47	4.71±1.75		41	4.54±1.27	
Yes	7	1.31±0.78		17	2.86±1.31		12	4.69±1.46	
Differentiation			*P*<0.01^b^,^1^			*P*<0.05^b^,^2^			p>0.05^b^
Normal	10	6.5±1.17		10	6.86±1.71		8	5.38±2.02	
I	8	6.23±1.72		19	5.01±2.03		16	4.98±1.53	
II	19	5.44±1.83		25	3.86±1.83		15	4.44±0.99	
III	16	4.95±2.23		20	3.92±1.44		22	4.40±1.43	
TNM stage			*P*<0.05^b^,^3^			*P*<0.01^b^			p>0.05^b^
Normal	10	6.50±1.17		10	6.86±1.71		8	5.38±2.02	
I	22	6.05±1.52		24	5.79±1.39		20	4.78±1.44	
II	7	4.77±1.16		19	4.02±0.93		33	4.47±1.31	
III	7	2.63±1.11		16	3.01±1.25		0	---	
IV	7	1.74±1.89		5	1.28±0.46		0	---	

a Student *t* test;

b one-way ANOVA

1 Differences between normal and grade I, II; grade I and II are not significant;

2 Difference between grade II and III is not significant;

3 Differences between normal and stage I; stage III and IV are not significant.

### FBXW7 regulates EMT in CCA cells

**Figure 2:**
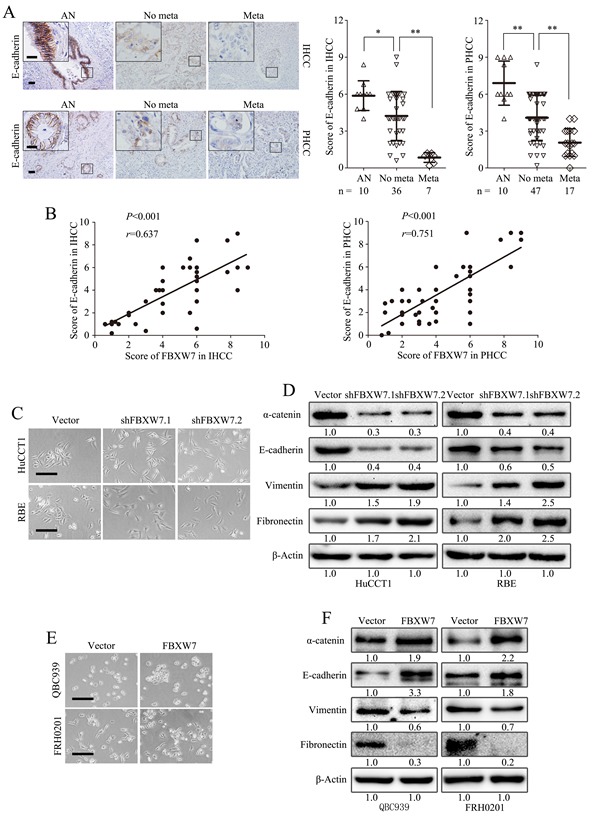
FBXW7 regulates EMT in CCA A. Representative images of E-cadherin IHC staining in IHCC and PHCC primary cancer tissues with or without metastasis and corresponding tumor adjacent nontumorous tissues. Corresponding semiquantification of E-cadherin expression was shown in right panels. AN, Tumor adjacent nontumorous tissue; No meta, primary cancers without metastasis (in situ);Meta, primary cancers with metastasis. B. Linear regression analyses of IHC scores between FBXW7 and E-cadherin in IHCC and PHCC respectively. C. Phase-contrast microscopic images of HuCCT1-shFBXW7 and RBE-shFBXW7 cells. D. Expression of epithelial markers (E-cadherin and α-catenin) and mesenchymal markers (Vimentin and Fibronectin) was analyzed by Western blotting. E. Phase-contrast images of QBC939-FBXW7 and FRH0201-FBXW7 cells. F. Expression of epithelial and mesenchymal markers was analyzed by Western blotting. Scale bars: 50μm (A) and 20μm (insets in A), 500μm (C and E). Numbers in (D and F) indicate the fold changes of band densities based on at least three independent experiments. * *P* < 0.05 and ** *P* < 0.01 based on the Student *t* test (A) or Spearman rank correlation test (B). Data are represented as mean ± SD.

IHC analysis of the tumor specimens exhibited a significant loss of E-cadherin expression in metastatic IHCC and PHCC (Fig. 2A) and a positive correlation between the expression of FBXW7 and E-cadherin in IHCC and PHCC (Fig. 2B), indicating FBXW7 may be involved in the EMT of CCA.

To elucidate the effects of FBXW7 on the EMT of CCA cells, we retrovirally established stable silencing of FBXW7 in HuCCT1 and RBE cells (designated as HuCCT1-shFBXW7.1, HuCCT1-shFBXW7.2, RBE-shFBXW7.1 and RBE-shFBXW7.2), and overexpression of FBXW7 in QBC939 and FRH0201 cells (designated as QBC939-FBXW7 and FRH0201-FBXW7). The FBXW7 expression levels in these resultant cell lines were verified by Western blotting and qRT-PCR (Supplementary Fig. S1C and D).

FBXW7 silenced cells showed morphological changes. HuCCT1-shFBXW7 and RBE-shFBXW7 cells displayed mesenchymal appearance and formed scattered colonies with reduced intercellular contacts, which are typical features of cells undergoing EMT (Fig. 2C). Western blotting revealed a clear loss of epithelial markers (E-cadherin and α-catenin) and increase of mesenchymal markers (Vimentin and Fibronectin) in HuCCT1-shFBXW7 cells and RBE-shFBXW7 cells (Fig. 2D).

Consistently, overexpression of FBXW7 in QBC939 and FRH0201 cells induced a more epithelial phenotype as compared with their control cells (Fig. 2E). Further analysis by Western blotting showed that overexpression of FBXW7 increased levels of epithelial markers (E-cadherin and α-catenin), accompanied by decreased levels of mesenchymal markers (Vimentin and Fibronectin) (Fig. 2F). Together, these observations suggest that FBXW7 is a regulator of EMT in CCA cells.

### FBXW7 inhibits migration and invasion of CCA cells

**Figure 3:**
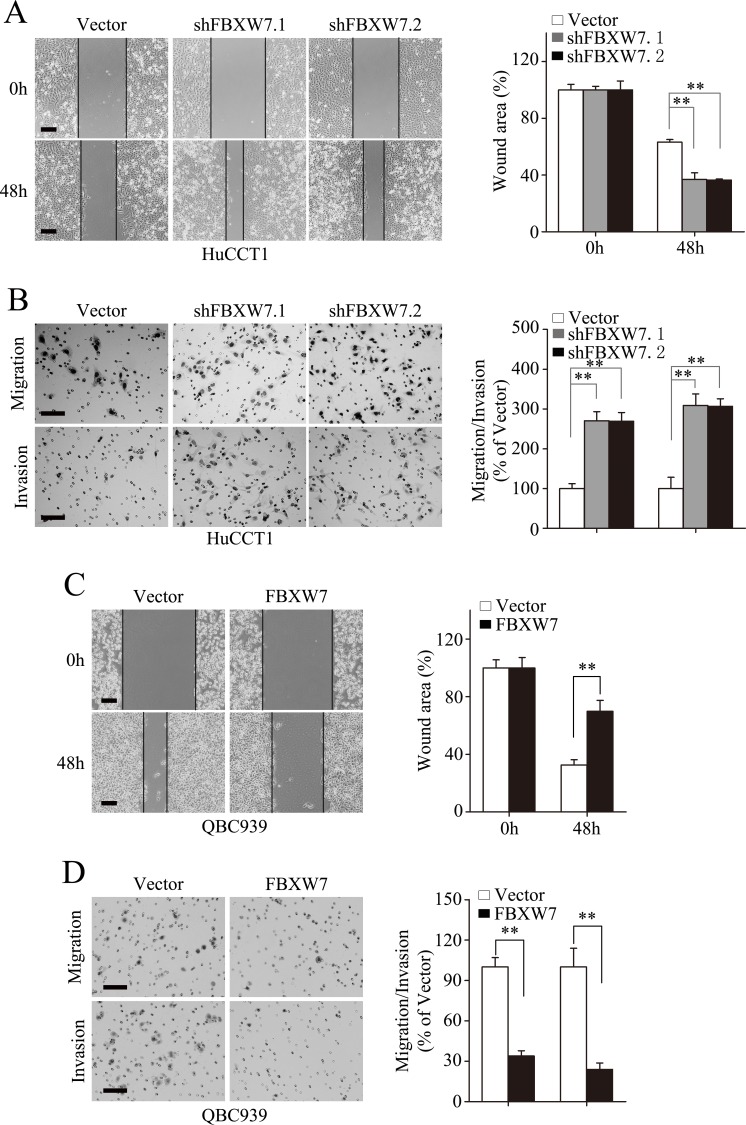
FBXW7 inhibits migration and invasion of CCA cells HuCCT1-shFBXW7 and QBC939-FBXW7 cells or control vector cells were subjected to wound healing assay (A and C), Transwell migration (B and D, top), and Matrigel invasion (B and D, bottom) assays. A. Quantification was carried out by measuring the uncovered areas compared with the controls. B. Quantification of migrated cells through the membrane (left columns) and invaded cells through matrigel (right columns) of each cell line are shown as proportions of their vector controls. C. Quantification was carried out by measuring the uncovered areas compared with the controls. D. Quantification of migrated cells through the membrane (left columns) and invaded cells through matrigel (right columns) of each cell line are shown as proportions of their vector controls. Scale bars: 500μm (A and C) and 50μm (B and D). ** *P* < 0.01 based on the Student *t* test. All results are from at least three independent experiments. Data are represented as mean ± SD.

To test the function of FBXW7 on EMT related cell behaviors in CCA cells, wound healing assay was firstly carried out, which showed markedly stimulated migration of the HuCCT1-shFBXW7 cells and RBE-shFBXW7 cells compared with their respective control cells (Fig. 3A; Supplementary Fig. S2A). This result was further confirmed by Boyden chamber assay (Fig. 3B, top panel; Supplementary Fig. S2B, top panel). In addition, HuCCT1-shFBXW7 and RBE-shFBXW7 cells showed a greater degree of invasion in Matrigel chamber assay (Fig. 3B, bottom panel; Supplementary Fig. S2B, bottom panel). Conversely, ectopic expression of FBXW7 markedly inhibited invasion and migration of QBC939 and FRH0201 cells (Fig. 3C and D; Supplementary Fig. S2C and D). These findings suggest that FBXW7 inhibits migratory and invasive potentials of CCA cells.

### FBXW7 attenuates emergence of stem cell-like characteristics in CCA cells

**Figure 4:**
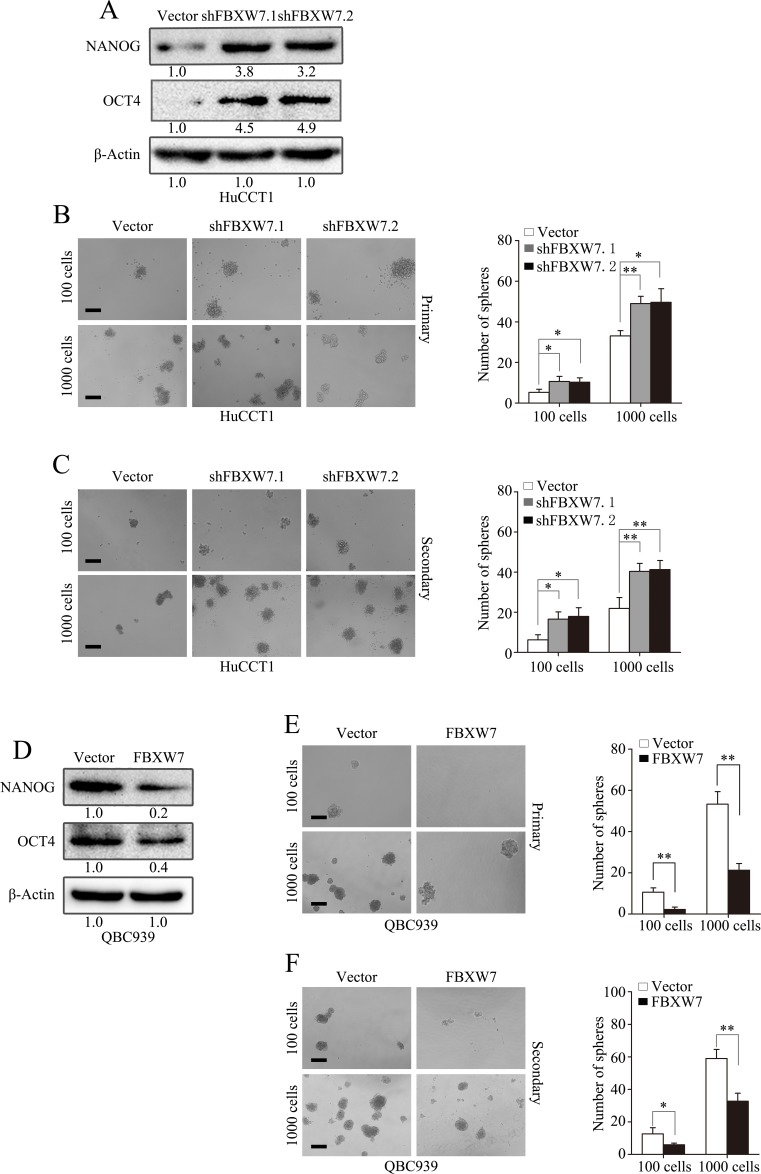
FBXW7 attenuates stem-like capacities of CCA cells A. Expression of cancer stem cell markers (NANOG and OCT4) were examined by Western blotting in HuCCT1-shFBXW7 cells and control vector cells. B. Number of spheres per well was quantified on primary spheroid formation assay for HuCCT1-shFBXW7 cells. Left panels showed representative spheres. C. Number of spheres per well was quantified on secondary spheroid formation assay for HuCCT1-shFBXW7 cells. Left panels showed representative spheres. D. Expression of cancer stem cell markers (NANOG and OCT4) were examined by Western blotting in QBC939-FBXW7 cells and control vector cells. E. Number of spheres per well was quantified on primary spheroid formation assay for QBC939-FBXW7 cells. Left panels showed representative spheres. F. Number of spheres per well was quantified on secondary spheroid formation assay for QBC939-FBXW7 cells. Left panels showed representative spheres. Scale bars: 50μm (B, C, E and F). Numbers in (A and D) indicate the fold changes of band densities based on at least three independent experiments. * *P* < 0.05 and ** *P* < 0.01 based on the Student *t* test. All results are from three independent experiments. Data are represented as mean ± SD.

Increasing evidences have illustrated a direct link between EMT and gain of stem cell like properties [13, 14]. Therefore, we investigated whether FBXW7 has an effect on the stem cell potential of CCA cells. Both HuCCT1-shFBXW7 and RBE-shFBXW7 cells exhibited significantly increased expression of OCT4 and NANOG relative to their control cells (Fig. 4A; Supplementary Fig. S3A). Consistently, the HuCCT1-shFBXW7 and RBE-shFBXW7 cells formed more tumor spheres on both primary and secondary spheroid formation assays (Fig. 4B and C; Supplementary Fig. S3B and C). In contrast, OCT4 and NANOG expression were significantly decreased in QBC939-FBXW7 and FRH0201-FBXW7 cells, accompanied with attenuated self-renewal ability as shown by primary and secondary spheroid formation assays (Fig. 4D-F; Supplementary Fig. S3D-F). Collectively, these results suggest that FBXW7 inhibits the cancer stem-like capacity of CCA cells.

### Rapamycin inhibits FBXW7 silencing-induced EMT and stem cell-like behavior in CCA cell lines

**Figure 5:**
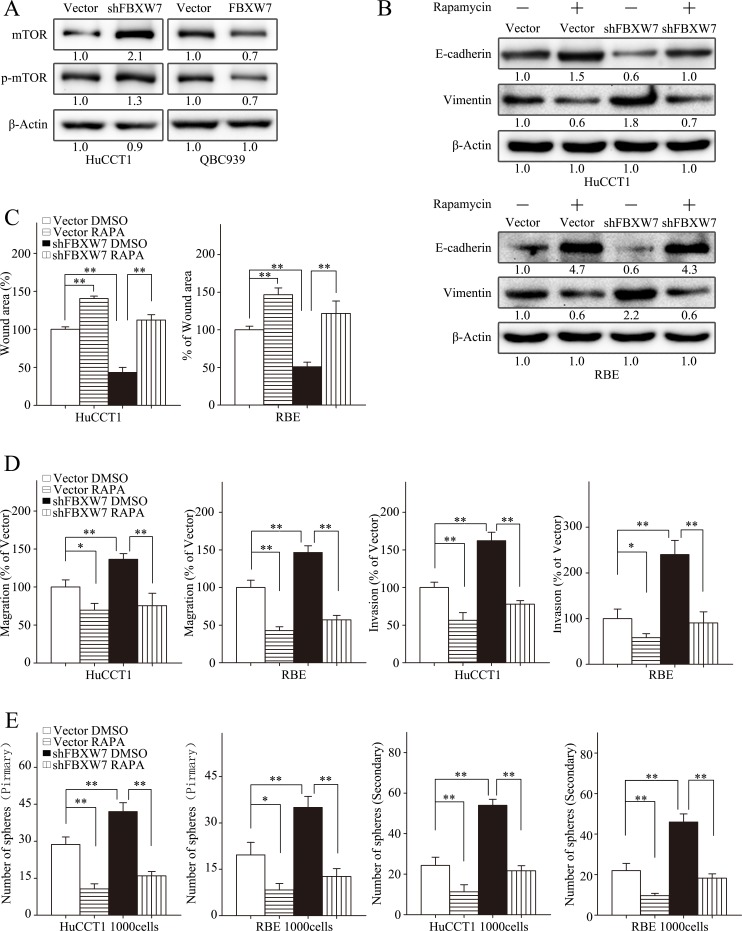
mTOR inhibitor rapamycin attenuates EMT, motility and stem-like behaviors induced by FBXW7silencing A. Total and phosphorylated mTOR levels of HuCCT1-shFBXW7, QBC939-FBXW7 cells or their vector control cells were analyzed by Western blotting. B-E. HuCCT1-vector, HuCCT1-shFBXW7, RBE-vector and RBE-shFBXW7 cells treated with rapamycin at 100nM or with DMSO were subjected to Western blotting (B), wound healing assay (C), Transwell migration assay (D, first two panels), Matrigel invasion assays (D, last two panels), primary and secondary spheroid formation assays (E). B. Expression of E-cadherin and Vimentin were analyzed by Western blotting. C and D. Quantification was carried out as described in Fig. 3. E. Quantification was carried out as described in Fig. 4. Numbers in (A and B) indicate the fold changes of band densities based on at least three independent experiments. * *P* < 0.05 and ** *P* < 0.01 based on the Student *t* test. All results are from three independent experiments. Data are represented as mean ± SD.

mTOR, one of FBXW7 downstream substrates, has been reported to involve in regulation of metastasis of CCA [15, 16]. To explore its role in mediating the function of FBXW7 in CCA, we first analyzed the relationship between FBXW7 and mTOR in CCA cells. Indeed, both total mTOR and p-mTOR were increased in HuCCT1-shFBXW7 cells in comparison with control cells, whereas QBC939-FBXW7 cells had lower levels of total mTOR and p-mTOR (Fig. 5A).

To confirm the role of mTOR in mediating the function of FBXW7 in regulating EMT, motility and stem-like characteristics of CCA cells, rapamycin, a mTOR inhibitor, was used. As expected, treatment with rapamycin significantly increased the expression of E-cadherin and decreased the expression of Vimentin in HuCCT1-shFBXW7 cells and RBE-shFBXW7 cells (Fig. 5B). Moreover, rapamycin significantly decreased migration and invasion of HuCCT1-shFBXW7 and RBE-shFBXW7 cells (Fig. 5C and D). Finally, both primary and secondary tumor sphere formation potentials were suppressed by rapamycin in HuCCT1-shFBXW7 and RBE-shFBXW7 cells (Fig. 5E). It is worth noting that FBXW7 silencing induced functional changes in migratory, invasive behaviors and tumor sphere formation capacities of CCA cells were eliminated by rapamycin treatment, and even more, HuCCT1-shFBXW7 and RBE-shFBXW7 cells behave much similar to HuCCT1 and RBE cells with rapamycin treatment (Fig. 5C-E). In addition, the *in vivo* metastasis of HuCCT1 cells induced by FBXW7 silencing was markedly suppressed with rapamycin treatment (Fig. 7A-C). IHC staining of the metastatic tumors also revealed an increased E-cadherin expression in tumors from HuCCT1-shFBXW7 cells with rapamycin treatment compared with tumors from HuCCT1-shFBXW7 cells without rapamycin treatment (Fig. 7F). Taken together, these data demonstrated that FBXW7 regulates cancer cell EMT, motility and stem-like characteristics possibly via mTOR signaling in CCA cells.

### ZEB1 mediates FBXW7/mTOR signaling induced EMT, migration, and invasion in CCA cells

To extend our understanding of the signaling pathways in regulating EMT of CCA cells by FBXW7, expression of Snail, Slug and ZEB1 were examined by Western blotting and qRT-PCR. Interestingly, the expression of these EMT regulators were significantly higher in FBXW7 silenced cells compared with HuCCT1 and RBE cells at both mRNA and protein levels (Supplementary Fig. S4A-D). Furthermore, the induction of Snail, Slug and ZEB1 caused by FBXW7 silencing was dramatically suppressed with rapamycin treatment (Supplementary Fig. S4A-D). These results indicate that Snail, Slug and ZEB1 may be the target genes of FBXW7/mTOR signaling pathway in regulating EMT of CCA cells.

To our knowledge, the functional roles of ZEB1 in EMT and metastasis have not been systematically investigated in CCA. Zhou et al. [17] have reported that the expression level of ZEB1 protein was higher in hepatocellular tumors tissues than that in the corresponding normal liver tissues, and ZEB1 high expression was correlated with hepatocellular carcinoma progression. We first analyzed the expression of ZEB1 in a panel of CCA cell lines. ZEB1 was highly expressed in CCA cell lines compared with HIBEpiCs by RT-PCR (Supplementary Fig. S5A, left) and Western blotting (Supplementary Fig. S5A, right).

**Figure 6:**
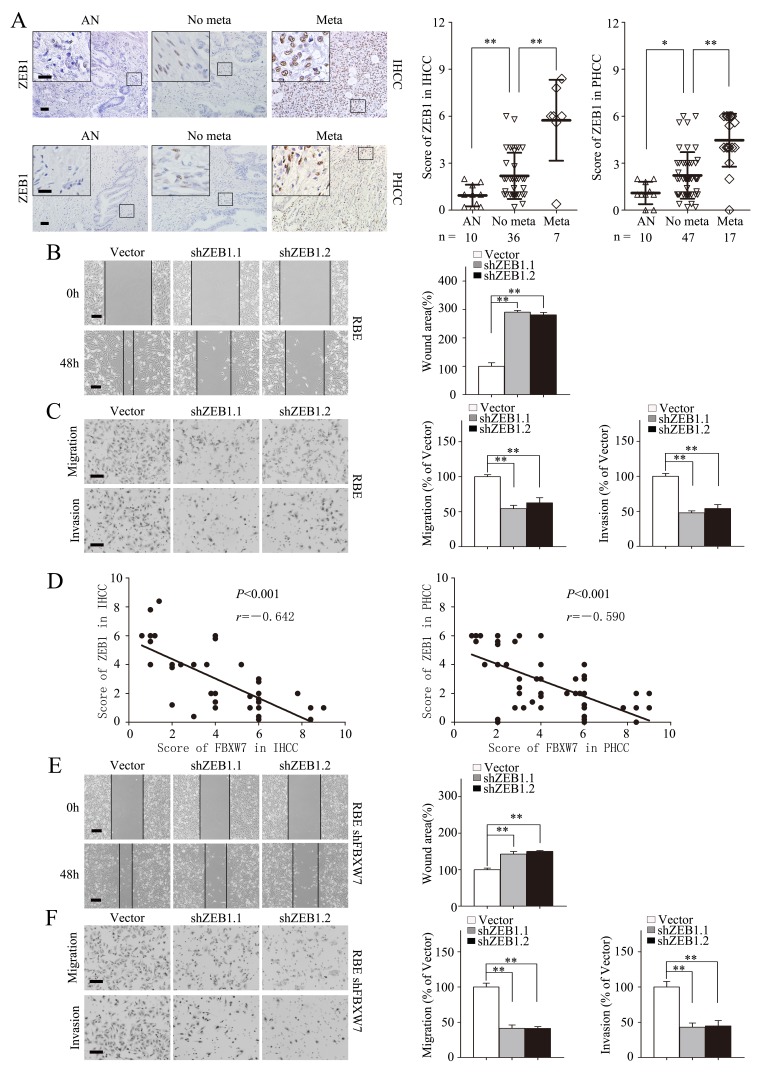
ZEB1 mediates FBXW7/mTOR signaling related EMT and metastasis in CCA A. Representative images of ZEB1 IHC staining in IHCC and PHCC primary cancer tissues with or without metastasis and corresponding tumor adjacent nontumorous tissues. Corresponding semiquantification of ZEB1 expression was shown in right panels. AN, Tumor adjacent nontumorous tissue; No meta, primary cancers without metastasis (in situ); Meta, primary cancers with metastasis. B and C. RBE cells transfected with shZEB1.1 or shZEB1.2 vector were subjected to wound healing assay (B), Transwell migration (C, top), and Matrigel invasion assays (C, bottom). Quantification was carried out as described in Fig. 3. D. Linear regression analyses of IHC scores between FBXW7 and ZEB1 in IHCC and PHCC respectively. E and F. RBE-shFBXW7 cells transfected with shZEB1.1 or shZEB1.2 vector were subjected to wound healing assay (E), Transwell migration (F, top), and Matrigel invasion (F, bottom) assays. Quantification was carried out as described in Fig. 3. Scale bars: 50μm (A, C and F) and 20μm (insets in A), 500μm (B and E). * *P* < 0.05 and ** *P* < 0.01 based on the Student *t* test (A-C, E and F) or Spearman rank correlation test (D). All results are from three independent experiments. Data are represented as mean ± SD.

Highly expressed ZEB1 in cancer tissues was also confirmed by IHC staining in 43 IHCC and 64 PHCC specimens as compared with adjacent nontumorous tissues, and was found positively correlated with IHCC and PHCC metastasis (Fig. 6A). Furthermore, the negative correlation between ZEB1 and E-cadherin expression levels was revealed by IHC staining in IHCC and PHCC (Supplementary Fig. S5B). Similarly, ZEB1 silencing by transient transfection with two distinct short hairpin RNAs (shRNA) in RBE significantly increased E-cadherin expression and decreased Vimentin expression (Supplementary Fig. S5C). Migration and invasion capacities were also decreased after silencing ZEB1 (Fig. 6B and C). Collectively, these data suggest that ZEB1 promotes EMT and metastasis in CCA.

To investigate the role of ZEB1 in FBXW7-related EMT and metastasis, we analyzed the phenotypic changes of the HuCCT1-shFBXW7 and RBE-shFBXW7 cells with ZEB1 knockdown. ZEB1 deficiency in these two cell lines led to increase in epithelial marker expression and decrease in mesenchymal marker expression at protein level (Supplementary Fig. S5D). A negative correlation between the expression of FBXW7 and ZEB1 was also confirmed in the above IHCC and PHCC specimens (Fig. 6D). Consistent with the phenotypic changes, the increased migratory and invasive capacities induced by FBXW7 silencing were reversed by silencing ZEB1 in both HuCCT1-shFBXW7 and RBE-shFBXW7 cell lines (Fig. 6E and F; Supplementary Fig. S5E and F). In conclusion, these findings suggest that ZEB1 mediates FBXW7 knockdown-induced EMT, migration, and invasion in CCA cells.

### FBXW7 regulates CCA cell EMT and metastasis *in vivo*

**Figure 7:**
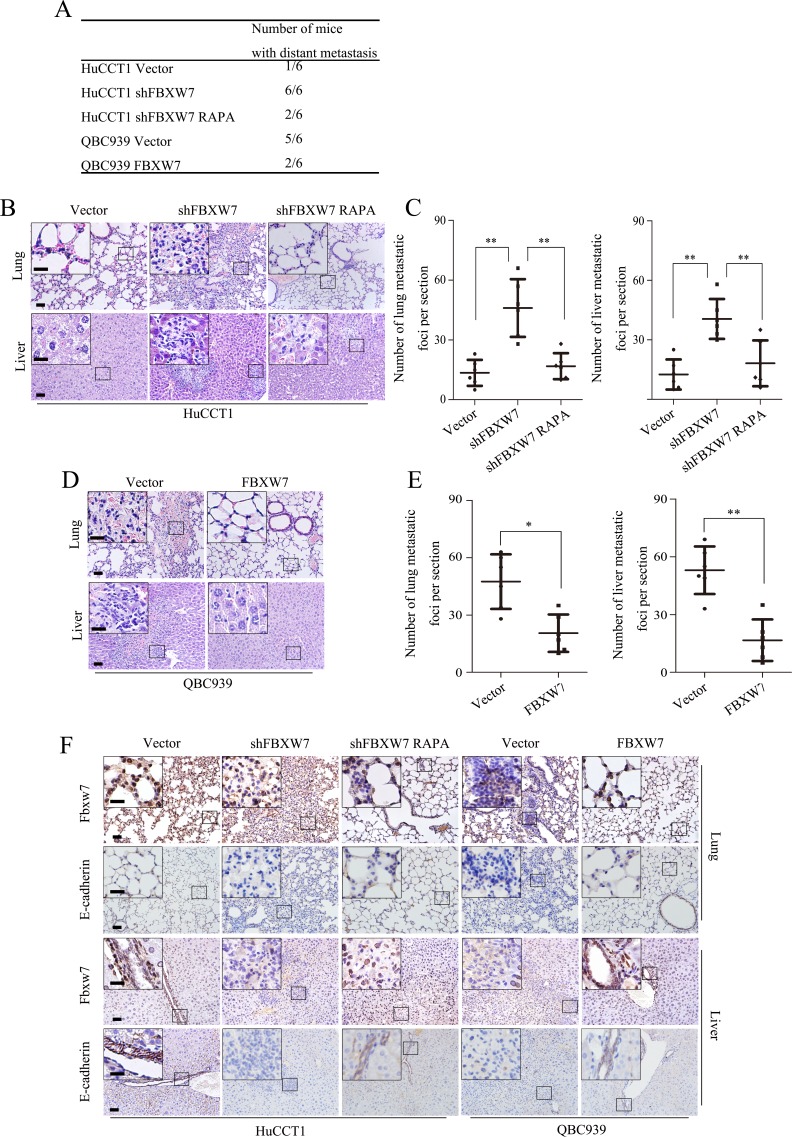
FBXW7 inhibits EMT and metastasis by regulating mTOR expression *in vivo* A. HuCCT1-vector, HuCCT1-shFBXW7, QBC939-vector and QBC939-FBXW7 cells were injected into 6 weeks old male nude mice intravenously via the tail vein (n = 6). Mice were sacrificed after 40 days, lung and liver tissues were harvested. The total numbers of mice with distant metastasis in every group were counted. B. Representative images of metastatic foci per section in lung (top) and liver (bottom) of individual mouse with injection of HuCCT1-shFBXW7 (with or without rapamycin treatment) or its control cells. C. Corresponding quantification of metastatic foci per section in (B). D. Representative images of metastatic foci per section in lung (top) and liver (bottom) of individual mouse with injection of QBC939-FBXW7 or its control cells. E. Corresponding quantification of metastatic foci per section in (D). F. Lung and liver sections were subjected to IHC analysis using FBXW7 and E-cadherin antibodies. Representative images are shown. Scale bars: 50μm (B, D and F) and 20μm (insets in B, D and F). * *P* < 0.05 and ** *P* < 0.01 based on the Student *t* test (C and E). Data are represented as mean ± SD.

To investigate whether FBXW7 regulates CCA metastasis *in vivo*, HuCCT1-shFBXW7, QBC939-FBXW7 and their control cells were injected into nude mice via the tail vein respectively. FBXW7 knockdown in HuCCT1 cells resulted in significant increase of the number of mice with distant metastasis (Fig. 7A) and the number of metastatic tumors in lung and liver of each mouse (Fig. 7B and C). Whereas, FBXW7 overexpression not only led to markedly decreased number of mice with distant metastasis (Fig. 7A), but also dramatically decreased the number of metastatic tumors in both lung and liver of each mouse (Fig. 7D and E). Consistent with our *in vitro* results, rapamycin treatment of the nude mice with HuCCT1-shFBXW7 injection significantly decreased FBXW7 knockdown-induced metastatic potential of CCA cells (Fig. 7A-C). We also investigated whether FBXW7 inhibited EMT of CCA cells *in vivo*. IHC staining analysis of the lung and liver sections revealed aggressive nature of the tumors from FBXW7 knockdown cells, as evidenced by reduced expression of E-cadherin, a hallmark of EMT, whereas the tumors from FBXW7 overexpression cells showed significantly increased expression of E-cadherin (Fig. 7F). These observations provid further evidence that FBXW7 is a potent inhibitor of CCA EMT and metastasis.

## DISCUSSION

In order to develop new prognostic markers and therapeutic targets for CCA, great efforts have been made to elucidate the molecular mechanisms underlying invasion and metastasis of CCA in the past several decades. Nevertheless, the detailed mechanisms of CCA metastasis remain obscure. FBXW7, a substrate recognition component of the SCF (complex of SKP1, CUL1 and F-box protein) complex, can bind to its substrates, which have been phosphorylated within conserved phosphor-degron motifs, and target them for ubiquitylation and subsequent degradation by the proteasome. It has been proven to mediate ubiquitin-dependent proteolysis of several well-known oncoproteins including Notch, cyclin E1 and mTOR, and regarded as a tumor suppressor at the crossroads of cell division, growth and differentiation [18]. Loss of FBW7 function leads to chromosomal instability, probably owing to hyperactivation of its many oncogenic substrates [16]. FBXW7 is regarded as a general tumor suppressor in human cancer. Mao et.al [19] reported that irradiation of FBXW7 heterozygotes increased incidences of tumors compared to wild-type animals. Furthermore, the loss of FBXW7 has been demonstrated to be related with poor prognosis in colorectal cancer, gastric cancer and IHCC [20-22]. Whereas, the functional roles of FBXW7 in EMT and cancer stem cell (CSC) characteristics of CCA is rarely reported, and the molecular pathways by which FBXW7 regulates EMT and CSC characteristics merit investigation.

In this report, we delineated for the first time the clinical significance of FBXW7 in both IHCC and PHCC and the mechanistic role of FBXW7 in regulating CCA metastasis. We found that the expression of FBXW7 was lower in both CCA cell lines and tumor tissues compared with normal intrahepatic bile duct epithelial cells and tumor adjacent tissues respectively. Clinical data analyses showed the lower expression of FBXW7 significantly correlated with metastasis, TNM stage and differentiation in both IHCC and PHCC, however, only a statistical trend was found in DCC, which may be due to the absence of more advanced (stage III and stage IV) tumor specimens in the dataset we analyzed (Table 1). Functionally, FBXW7 silencing in CCA cells induced EMT, CSC behaviors and motility of CCA cells *in vitro* and accelerated metastasis *in vivo*. Conversely, FBXW7 overexpression reversed these events both *in vitro* and *in vivo*. Mechanistically, our data indicated a potential link between FBXW7 and ZEB1 through FBXW7-mediated proteolysis of mTOR. These observations confirmed the crucial role of FBXW7 in CCA EMT and metastasis, and suggest that FBXW7 may serve as a potential molecular marker for CCA treatment and prognosis prediction.

EMT plays crucial roles during embryonic development and tissue repair, but it can adversely promote carcinoma progression through endowing cells with migratory and invasive properties and CSC-like phenotype, which may be prerequisites for cancer cell metastasis [9, 23, 24]. Thus, induction of EMT is considered to be an important and central mechanism for the progression of carcinomas to a metastatic stage and the maintenance of malignancy [25]. To our knowledge, no previous study has explored the relationship between FBXW7 and EMT regulators. As is shown in Supplementary Fig. S4, the expression of EMT regulators, including ZEB1, Snail and Slug, are significantly higher in FBXW7 silenced cells compared with corresponding control cells at both mRNA and protein levels. Furthermore, rapamycin can dramatically suppress the induction of EMT regulators caused by FBXW7 silencing, which indicates the potential role of mTOR in the FBXW7 mediated expression of EMT regulators. In dissecting the molecular mechanisms underlying the EMT regulation by FBXW7/mTOR, we focused on ZEB1 as its function and clinical significance in CCA remain undeclared although Mizuguchi et.al [26] have reported ZEB1 may be involved in SPRR2a regulated EMT. In this study, we revealed a positive correlation between ZEB1 and CCA metastasis and confirmed that FBXW7 silencing significantly increased ZEB1 expression and rapamycin treatment dramatically reversed this FBXW7 silencing induced ZEB1 expression in CCA cells, implicating that ZEB1 might be an important downstream target of FBXW7/mTOR signaling. Although Mikaelian. et. al [27] have reported that miR-200 miRNAs were involved in the process of mTOR mediated ZEB1 expression, the detailed mechanisms regarding how FBXW7/mTOR regulating ZEB1 expression in CCA need further studies.

mTOR, an ubiquitination target of FBXW7 [16], is regarded as potential therapeutic target of CCA in recent reports [15, 28, 29]. Most of recent studies have supported a function for elevated mTOR activity in promoting EMT [30-32]. However, Mikaelian. et. al [27] demonstrated that mTOR could maintain the epithelial phenotype by inhibiting EMT. In this study, mTOR inhibition with rapamycin dramatically blocked the EMT, CSC behaviors and cell motility of CCA cells *in vitro* and metastasis *in vivo* induced by FBXW7 silencing, supporting a fundamental role of mTOR inhibitor in inhibiting FBXW7 deficiency induced metastasis of CCA.

Therapeutically, rapamycin mediated inhibition of mTOR resulted in loss of leukemia-initiating stem cells and gain of normal hematopoietic stem cells in leukemia [33]. Inhibition of mTOR signaling may prevent CSC self-renewal and circumvent CSC-mediated resistance to cancer therapeutics [23]. Previous studies have demonstrated that mTOR inhibitor, combined with other antitumor agents or not, could inhibit the development of CCA [28, 29, 34, 35]. ZEB1 silencing, either chemically or by RNAi, in mesenchymal-like cells results in a partial epithelial metaplasia and drug sensitivity [36, 37]. Considering the positive correlation between mTOR and ZEB1 in CCA demonstrated in this study, mTOR inhibitor may be a potential therapeutic strategy to increase the chemotherapy sensitivity and effectiveness of CCA. Liu et.al [38] revealed temporal mTOR inhibition protected Fbxw7-deficient mice from radiation-induced tumor development. Of note, Liza C. et.al [39] reported a lung adenocarcinoma patient with FBXW7 deficiency both clinically and radiographically benefited from treatment with the mTOR inhibitor. Thus, the results observed in this study, together with previous reports, underlie mTOR inhibitor as a promising strategy to be added to CCA chemotherapy regimen, especially for patients with FBXW7 deficiency.

In conclusion, our study highlights a pivotal role for FBXW7 in inhibiting EMT and metastasis in CCA via regulating the mTOR/ZEB1 signaling pathway. Our observations that rapamycin dramatically blocked the tumor metastasis induced by silencing FBXW7 both *in vitro* and *in vivo* provided us a therapeutic option by targeting mTOR in FBXW7 deficient patients in clinical practice. Furthermore, our results suggest that FBXW7 may serve as a potential molecular marker for CCA treatment and prognosis.

## MATERIALS AND METHODS

### Cell lines and cell culture

The human intrahepatic biliary epithelial cell line HIBEpiC, IHCC cell line RBE, PHCC cell line QBC939 and FRH0201 were purchased from Cell Bank of the Chinese Academy of Sciences (Shanghai, China). IHCC cell line HuCCT1 was purchased from RIKEN Bioresourse Center (Koyadai, Japan). HEK 293 Phoenix ampho packaging cells were purchased from the American Type Culture Collection. All cell lines were cultured in DMEM medium or RPMI 1640 medium supplemented with 10% fetal bovine serum and penicillin/streptomycin. All cells were used no more than 6 months after purchase for the experiments described herein.

### Clinical specimen collection

One hundred and sixty patients (including 43 IHCC patients, 64 PHCC patients and 53 DCC patients) underwent surgical resection at Qilu Hospital of Shandong University (Jinan, China) between January 2005 and October 2012 were included in this study. Tumors (n = 160) and tumor adjacent tissues (n = 28) were obtained from curative resection. Seven fresh paired CCA and adjacent normal bile duct tissues were obtained from CCA patients undergoing curative surgical resection of the primary tumor from March to October 2013 at Qilu Hospital of Shandong University. Tissue samples were kept at −180°C liquid nitrogen freezers before use. Final pathologic diagnosis of all the specimens was confirmed by pathologists in Qilu Hospital of Shandong University. Pathologic tumor-node-metastasis (pTNM) staging is based on the 7th staging classification of AJCC/UICC (2010). The experimental protocols were approved by patients' signed consent and the institutional review committee.

### Immunohistochemistry and its scoring

The IHC analysis of FBXW7, ZEB1 and E-cadherin (Supplementary Table 2) expression in clinical samples was performed as previously described [40]. All slides were scored by two pathologists blinded to the pathology and clinical features at Qilu Hospital of Shandong University. The scoring system included the extent and intensity of staining as described elsewhere [41]. The score for the extent of the IHC stained area was scaled as 0 for no IHC signal at all, 1 for less than 10%, 2 for 10%-50%, and 3 for more than 50% of cells stained. The score for IHC intensity was also scaled as 0 for no IHC signal, 1 for weak, 2 for moderate, and 3 for strong IHC signals. Five randomly selected high-power fields (×400 magnification) were photographed for each IHC slide. The overall quantitation of IHC score was obtained by multiplying the average intensity and extent score of the five different high-power fields, with a maximum score of 9.

### *In vivo* studies

Male athymic nude mice (6 weeks old) were purchased from Institute of Laboratory Animal Science, Chinese Academy of Medical Science and Peking Union Medical College (CAMS & PUMC) and maintained in microisolator cages. All animals were used in accordance with institutional guidelines and the current experiments were approved by the Use Committee for Animal Care. For metastasis assays, cells were resuspended in PBS at a concentration of 2×10^7^ cells/mL. A volume of 0.1 mL of suspended HuCCT1-shFBXW7 (with or without rapamycin treatment), QBC939-FBXW7 and their control cells was injected into the tail veins (6 mice per group). Rapamycin (R8781, Sigma) treated HuCCT1-shFBXW7 group were injected with rapamycin intraperitoneally at 2.5mg/kg/d for 2 consecutive weeks after tail vein injection. All of the mice were sacrificed by CO_2_ 40 days after inoculation. Lung and liver were dissected out, fixed in 10% buffer formalin and paraffin embedded. Serial sections of lung and liver tissues were made and examined by hematoxylin and eosin (H&E) staining and IHC staining.

### Statistical analysis

Results are presented as mean ± standard deviation (SD). Student *t*-test and one-way ANOVA were used where indicated. Linear regressions were tested by using the Spearman rank correlation. All statistical tests were two-sided and *P* values were considered statistically significant for *P* < 0.05. Statistical analysis was carried out with SPSS 17.0 software (SPSS Inc.).

## SUPPLEMENTARY MATERIALS AND METHODS FIGURES AND TABLES


