# Supplementary material for: FBXW7 suppresses epithelial-mesenchymal transition, stemness and metastatic potential of cholangiocarcinoma cells
Source: Oncotarget. 2015 Jan 31;6(8):6310–25. doi: 10.18632/oncotarget.3355 (PMC4467439; doi:10.18632/oncotarget.3355)
Supplement: Supplementary file 1 [file oncotarget-06-6310-s001.pdf]

FBXW7 suppresses epithelial-mesenchymal transition, stemness and metastatic potential of cholangiocarcinoma cells

Supplementary Materials and Methods

Western blotting analysis

To measure protein levels, cell lysates were separated by reducing SDS-PAGE before electrotransfering to PVDF membrane. Blocked membranes were incubated with the primary antibodies (Supplementary Table 2). The signals from the primary antibody was amplified by HRP conjugated anti-mouse IgG (1:10000 dilution) or anti-rabbit IgG (1:10000 dilution), the bands was visualized by FluorChem E system (Protein Simple, USA).

Reverse transcription–polymerase chain reaction (RT–PCR)

Total RNA from cultured cells was extracted using TRIzol reagent (15596-026, Invitrogen), and 5 μg of RNA was used for cDNA synthesis. Quantitative real-time PCR (qRT-PCR) and data collection were performed with an ABI PRISM 7900HT sequence detection system with the preset PCR program and GAPDH as an internal control. For semiquantitative RT-PCR, the products were analyzed by agarose gel electrophoresis. The sequences of primers are detailed in Supplementary Table 1.

Establishment of FBXW7 stable expression and FBXW7/ZEB1 knockdown cell lines

pBabe.puro retroviral construct containing human FBXW7 cDNA, two shRNA against human FBXW7 expressed in pBabe.puro vector and two shRNA against human ZEB1expressed in pSingle-tTS vector were prepared as described previously [1]. The generation of retrovirus supernatants and transfection of cholangiocarcinoma cells were conducted as described previously [1]. Infected cells were selected by adding 2μg/ml puromycin to the culture medium for 48 h and then maintained in complete medium with 0.5μg/ml puromycin. Empty retroviral-infected stable cell lines were also produced by the above protocols. The expression of FBXW7 was confirmed by Western blotting and qRT–PCR analysis. The tight on/off regulation of the pSingle-tTS vector system and coordinate inactivation of the target gene was mediated using doxycycline.

Wound healing assay

For each test, cells were grown to confluence in a 6-well plate, and were wounded with a 200μl sterile pipette tips. The wounded area was photographed using phase-contrast microscopy at 0h and 48h. Quantification, from at least three separate experiments, was carried out by measuring the uncovered areas compared with the controls.

Migration and invasion assays

A Boyden chamber (8μm pore size, BD Biosciences, USA) was used for *in vitro* migration and invasion assays. For migration assay, cells (5×10^4) in 200μl of serum-free medium were plated in the upper chamber, whereas 600μl of medium with 10% fatal bovine serum were added to lower well. After 24 h incubation, the cells were removed from the upper side of the chamber, and cells migrated to the lower surface of the membrane were fixed and stained. For each membrane, five random fields were counted at ×10 magnification. The mean cell number was calculated and data was presented as mean ± SD from three independent experiments done in triplicate. The invasion assay was conducted similarly to the migration assay except that transwell chambers were precoated with 50μl of 1mg/ml Matrigel (BD Biosciences, USA) matrix according to the manufacturer's recommendations. The incubation time was 48 h for invasion assay.

Spheroid formation assay

The capability of self-renewal was assessed using ultralow attachment 96-well-plate (Corning, USA). Different number of cells (100 cells/well or 1000 cells/well) were seeded and incubated in a cell culture incubator for 1 week in McCoy's 5A supplemented with serum free medium and phase-contrast images were obtained. For secondary spheres, spheres were enzymatically dissociated (Trypsin-EDTA), and seeded as density of 100 cells/well or 1000 cells/well, then cultivated for another 7 d.

References

1. Wang Y, Wen M, Kwon Y, Xu Y, Liu Y, Zhang P, He X, Wang Q, Huang Y, Jen KY, LaBarge MA, You L, Kogan SC, Gray JW, Mao JH and Wei G. CUL4A induces epithelial-mesenchymal transition and promotes cancer metastasis by regulating ZEB1 expression. Cancer research. 2014; 74(2):520-531.

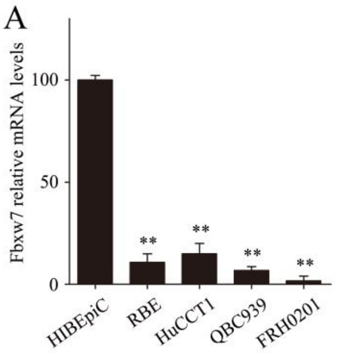

B

Table The clinicopathological characteristics of the 7 pairs of CCA fresh tissues

	Gender [a]	Age [b]	Type [c]	Tumor size [d]	TNM [e]	Differentiation [f]	Metastasis [g]
1	M	61	PHCC	3	II	P	N
2	F	57	PHCC	2.5	I	M	N
3	M	61	PHCC	2	III	M	Y
4	M	55	PHCC	2.3	II	W	N
5	M	71	PHCC	3	III	M	Y
6	F	64	PHCC	5	III	P	Y
7	F	57	PHCC	4	II	W	N

a, M: Male, F: Female; b, Age: years; c, PHCC: perihilar cholangiocarcinoma;
d, Tumor size: diameter (cm); e, TNM staging is based on the 7th staging classification of AJCC/UICC (2010); f, Differentiation: W: Well, M: Moderately,P: Poorly;
g, Metastasis: N: without metastasis, Y: with metastasis.

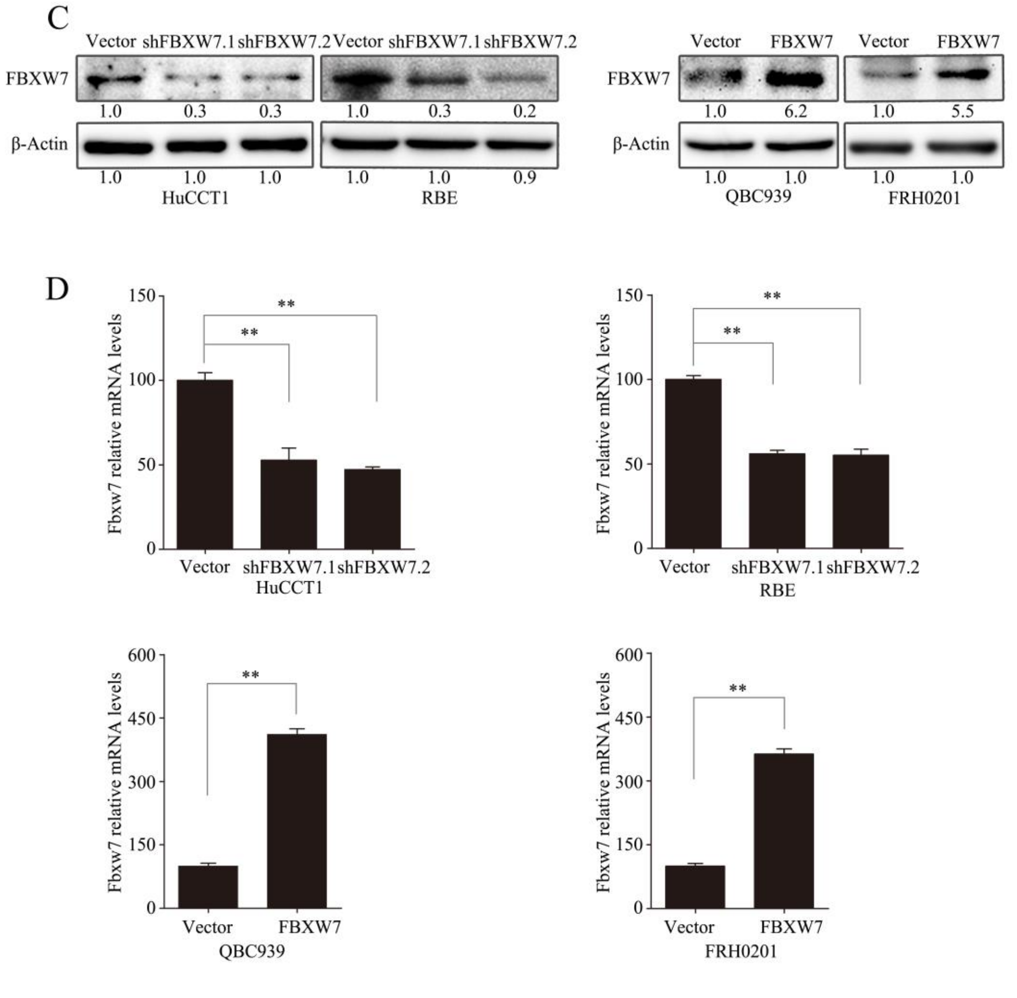

Supplemental Figure 1: FBXW7 is downregulated in CCA. A. FBXW7 mRNA levels in HIBEpiC and four human CCA cell lines were examined by qRT - PCR. B. Clinicopathological characteristics of 7 pairs of CCA fresh tissues. C and D.

Validation of FBXW7 protein and mRNA levels by Western blotting (C) and qRT-PCR (D) in four human CCA cell lines after stable viral transfection. Numbers in (C) indicate the fold changes of band densities based on at least three independent experiments. ** $P < 0.01$ based on the Student t test. All results are from at least three independent experiments. Data are represented as mean ± SD.

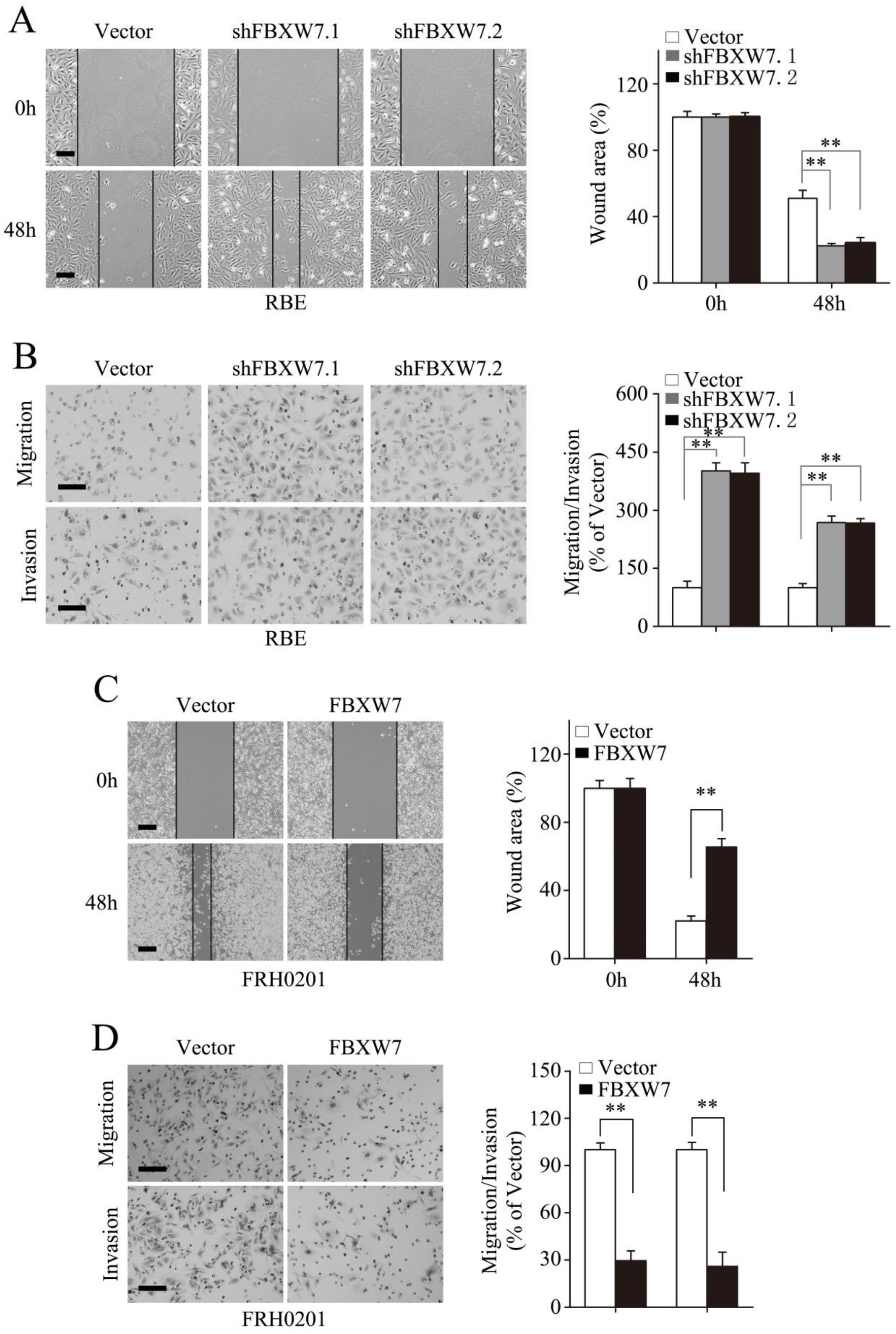

Supplemental Figure 2: FBXW7 inhibits migration and invasion of CCA cells.

RBE-shFBXW7 and FRH0201-FBXW7 cells or control vector cells were subjected to

wound healing assay (A and C), Transwell migration (B and D, top), and Matrigel invasion (B and D, bottom) assays. A. Quantification was carried out by measuring the uncovered areas compared with the controls. B. Quantification of migrated cells through the membrane (left columns) and invaded cells through matrigel (right columns) of each cell line are shown as proportions of their vector controls. C. Quantification was carried out by measuring the uncovered areas compared with the controls. D. Quantification of migrated cells through the membrane (left columns) and invaded cells through matrigel (right columns) of each cell line are shown as proportions of their vector controls. Scale bars: 500μm (A and C) and 50μm (B and D). ** $P < 0.01$ based on the Student *t* test. All results are from at least three independent experiments. Data are represented as mean ± SD.

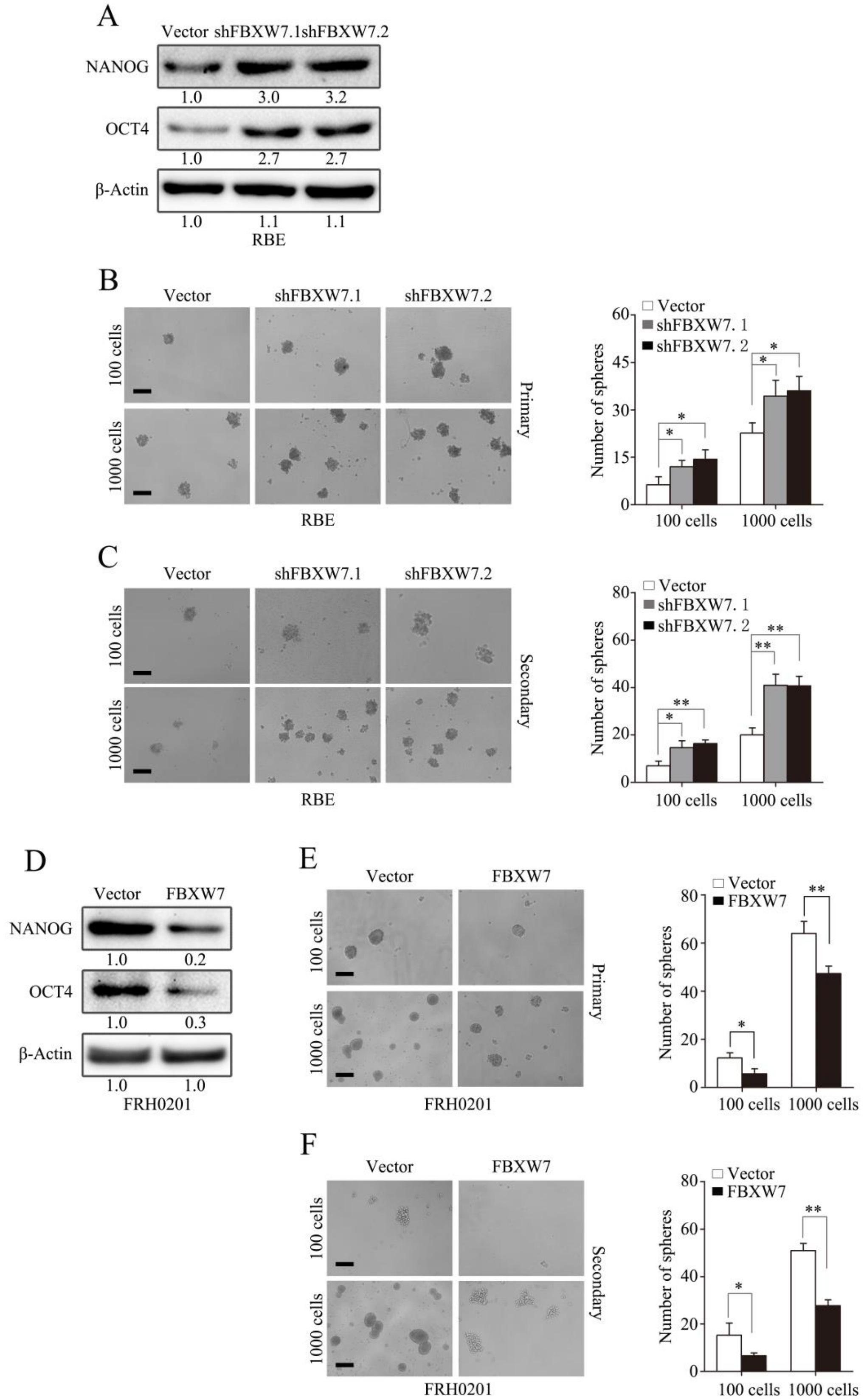

A
Vector shFBXW7.1 shFBXW7.2
NANOG
1.0 3.0 3.2
OCT4
1.0 2.7 2.7
β-Actin
1.0 1.1 1.1
RBE
B
Vector shFBXW7.1 shFBXW7.2
100 cells
1000 cells
Primary
RBE
Vector
shFBXW7. 1
shFBXW7. 2
Number of spheres
60
45
30
15
0
100 cells 1000 cells
C
Vector shFBXW7.1 shFBXW7.2
100 cells
1000 cells
Secondary
RBE
Vector
shFBXW7. 1
shFBXW7. 2
Number of spheres
80
60
40
20
0
100 cells 1000 cells
D
Vector FBXW7
NANOG
1.0 0.2
OCT4
1.0 0.3
β-Actin
1.0 1.0
FRH0201
E
Vector FBXW7
100 cells
1000 cells
Primary
FRH0201
Vector
FBXW7
Number of spheres
80
60
40
20
0
100 cells 1000 cells
F
Vector FBXW7
100 cells
1000 cells
Secondary
FRH0201
Vector
FBXW7
Number of spheres
80
60
40
20
0
100 cells 1000 cells

Supplemental Figure 3: FBXW7 attenuates stem-like capacities of CCA cells.

A. Expression of cancer stem cell markers (NANOG and OCT4) were examined by Western blotting in RBE-shFBXW7 cells and control vector cells. B. Number of spheres per well was quantified on primary spheroid formation assay for RBE-shFBXW7 cells. Left panels showed representative spheres. C. Number of spheres per well was quantified on secondary spheroid formation assay for RBE-shFBXW7 cells. Left panels showed representative spheres. D. Expression of cancer stem cell markers (NANOG and OCT4) were examined by Western blotting in FRH0201-FBXW7 cells and control vector cells. E. Number of spheres per well was quantified on primary spheroid formation assay for FRH0201-FBXW7 cells. Left panels showed representative spheres. F. Number of spheres per well was quantified on secondary spheroid formation assay for FRH0201-FBXW7 cells. Left panels showed representative spheres. Scale bars: 50μm (B, C, E and F). Numbers in (A and D) indicate the fold changes of band densities based on at least three independent experiments. * $P < 0.05$ and ** $P < 0.01$ based on the Student *t* test. All results are from three independent experiments. Data are represented as mean ± SD.

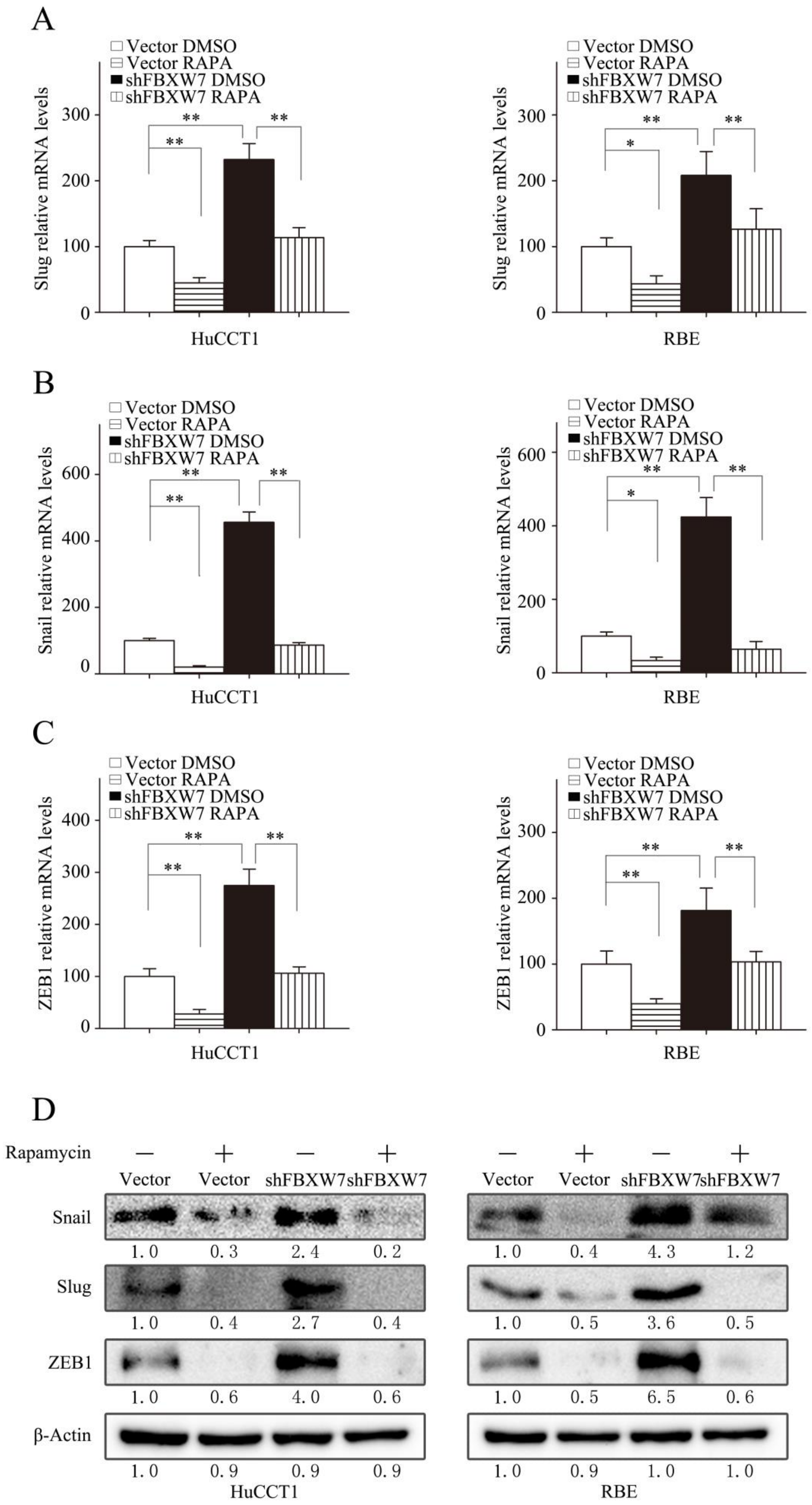
A
Vector DMSO
Vector RAPA
shFBXW7 DMSO
shFBXW7 RAPA
Slug relative mRNA levels
HuCCT1
RBE
B
Snail relative mRNA levels
HuCCT1
RBE
C
ZEB1 relative mRNA levels
HuCCT1
RBE
D
Rapamycin
Vector
shFBXW7
Snail
1.0 0.3 2.4 0.2
1.0 0.4 4.3 1.2
Slug
1.0 0.4 2.7 0.4
1.0 0.5 3.6 0.5
ZEB1
1.0 0.6 4.0 0.6
1.0 0.5 6.5 0.6
β-Actin
1.0 0.9 0.9 0.9
1.0 0.9 1.0 1.0
HuCCT1
RBE

Supplemental Figure 4: EMT transcriptional regulator ZEB1, Snail and Slug are involved in FBXW7/mTOR signaling pathway in CCA cells. A-D. HuCCT1-vector, HuCCT1-shFBXW7, RBE-vector and RBE-shFBXW7 cells with or without rapamycin treatment were subjected to qRT-PCR (A-C) and Western blotting (D) analysis to examine the mRNA and protein levels of ZEB1, Snail and Slug, respectively. Numbers in (D) indicate the fold changes of band densities based on at least three independent experiments. * $P < 0.05$ and ** $P < 0.01$ based on the Student *t* test. All results are from three independent experiments. Data are represented as mean ± SD.

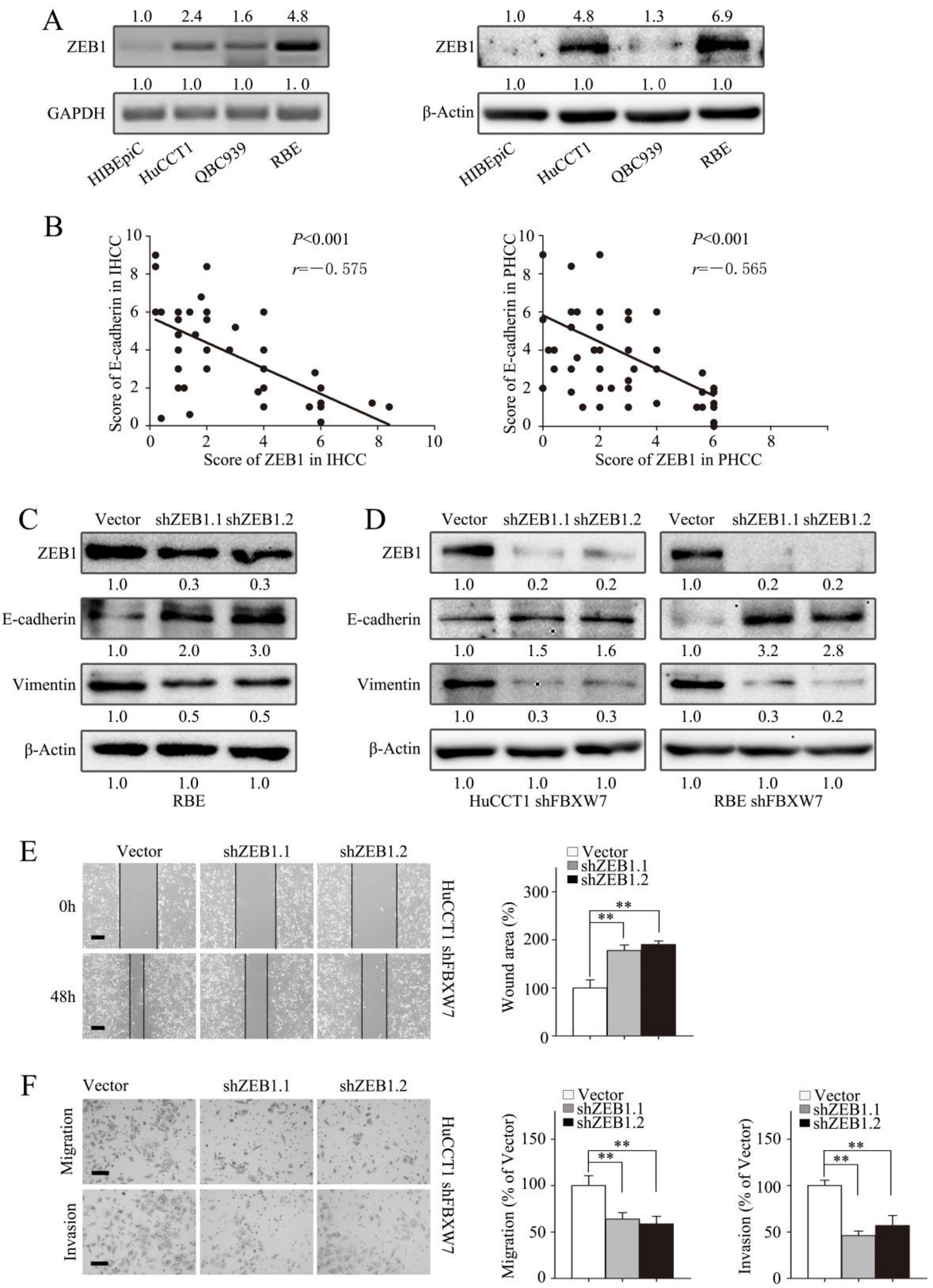

A
ZEB1
GAPDH
HIBEpiC
HuCCT1
QBC939
RBE
β-Actin
B
P<0.001
r=−0.575
r=−0.565
Score of E-cadherin in IHCC
Score of ZEB1 in IHCC
Score of E-cadherin in PHCC
Score of ZEB1 in PHCC
C
D
Vector
shZEB1.1
shZEB1.2
E-cadherin
Vimentin
HuCCT1 shFBXW7
RBE shFBXW7
E
0h
48h
Wound area (%)
F
Migration
Invasion
Migration (% of Vector)
Invasion (% of Vector)

Supplemental Figure 5: ZEB1 mediates FBXW7 related EMT and metastasis in CCA cells. A. ZEB1 expression was detected in HIBEpiC and three human CCA cell lines by RT-PCR (left) and Western blotting (right). B. Linear regression analysis of IHC scores between ZEB1 and E-Cadherin in IHCC and PHCC respectively. C. Western blotting was applied to show E-Cadherin and Vimentin expression in RBE cells after downregulation of ZEB1. D. Silencing ZEB1 restored E-Cadherin expression and decreased Vimentin expression in both HuCCT1-shFBXW7 and RBE-shFBXW7 cells. E and F. HuCCT1-shFBXW7 cells transfected with shZEB1.1 vector or shZEB1.2 vector were subjected to wound healing assay (E), Transwell migration (F, top), and Matrigel invasion (F, bottom) assays. Quantification was carried out as described in Fig. 3. Scale bars: 500μm (E) and 50μm (F). Numbers in (C and D) indicate the fold changes of band densities based on at least three independent experiments. ** $P < 0.01$ based on the Student *t* test (E and F) or Spearman rank correlation test (B). Data are represented as mean ± SD.

Supplementary Table S1: Oligonucleotide Sequences of RT-PCR Primers

**Gene symbol**	**Forward sequence (5'-3')**	**Reverse sequence (5'-3')**
FBXW7	AGCTGGAGTGGACCAGAGAAATTG	GCTGCTTGTAGCAGGTCTTTGGGT
Slug	TTCGGACCCACACATTACCT	GCAGTGAGGGCA AGA AAAAG
Snail	ATCGGAAGCCTAACTACAGCGA	CACGCCTGGCACTGGTACTTCT
ZEB1	AGCAGTGAAAGAGAAGGG AATGC	GGT CCT CTTCAGGTGCCTCAG
GAPDH	GCCGCATCTTCTTTTGCGTCGC	TCCCGTTCTCAGCCTTGACGGT

Supplementary Table S2: The Types, Dilutions and Sources of Antibodies Used for Western Blotting and Immunohistochemical Analysis

Antibody	Working dilution Western blotting	Working dilution IHC	Species	Source -Cat. Number
FBXW7	1:1000	1:300	Rabbit polyclonal	Abcam (Cat. No.ab109617)
mTOR	1:1000	—	Rabbit monoclonal	Cell Signaling Technology (Cat. No.2983)
p-mTOR	1:1000	—	Rabbit polyclonal	Cell Signaling Technology (Cat. No. 2971)
ZEB1	1:3000	1:150	Mouse polyclonal	Cell Signaling Technology (Cat. No. ab180905)
E-Cadherin	1:1000	1:400	Rabbit monoclonal	Cell Signaling Technology (Cat. No. 24E10)
Vimentin	1:1000	—	Rabbit polyclonal	Cell Signaling Technology

				(Cat. No. 3932)
α-Catenin	1:100	—	Mouse monoclonal	BD Biosciences (Cat. No. 610193)
Fibronectin	1:1000	—	Rabbit polyclonal	Abcam (Cat. No. ab2413)
Nanog	1:1000	—	Mouse monoclonal	Cell Signaling Technology (Cat. No.4893)
OCT4	1:1000	—	Rabbit monoclonal	Cell Signaling Technology (Cat. No. NB110-91359)
snail	1:1000	—	Rabbit monoclonal	Cell Signaling Technology (Cat. No. 3879)
slug	1:1000	—	Mouse monoclonal	Cell Signaling Technology (Cat. No. 9589)
β-Actin	1:2000	—	Rabbit monoclonal	Cell Signaling Technology (Cat. No. 8457)
